# The role of the gut microbiota during the first 2 years of life in the early programming of obesity, type 2 diabetes, and hypertension

**DOI:** 10.3389/fnut.2026.1772889

**Published:** 2026-03-11

**Authors:** Ana Lizette Rojas-Rodríguez, Valentina Jaramillo-Romero

**Affiliations:** Departamento de Ciencias de la Salud, Universidad Técnica Particular de Loja, Loja, Ecuador

**Keywords:** gastrointestinal microbiome, infant nutrition, obesity, type 2 diabetes mellitus, hypertension, early-life programming, cardiometabolic programming

## Abstract

The first 2 years of life constitute a critical window for the establishment of the gut microbiota and the early programming of cardiometabolic risk. The aim of this review was to analyze the influence of the gut microbiota during the first 2 years of life and its association with obesity, type 2 diabetes, and arterial hypertension. The reviewed studies suggest that early dysbiosis is associated with increased cardiometabolic vulnerability, linked to low-grade inflammation and alterations in energy metabolism. Associations are described between maternal metabolic conditions (such as obesity or gestational diabetes) and a less favorable initial intestinal ecosystem in the child, characterized by lower microbial diversity and reduced abundance of bacteria considered protective. In childhood obesity, longitudinal studies indicate that less mature microbiomes during the first year of life are associated with a higher risk of overweight, particularly when early antibiotic exposure and unhealthy dietary patterns coexist. In contrast, exclusive breastfeeding is associated with more functional microbial profiles. Regarding arterial hypertension, the findings suggest an influence mediated by microbial metabolites such as short-chain fatty acids and mechanisms involved in vascular regulation. Overall, the first 1,000 days represent a priority axis for promoting early-life practices that support a balanced gut microbiota as a potential strategy for cardiometabolic disease prevention.

## 1 Introduction

During the first 1,000 days of life, from conception to 2 years of age, humans undergo a key developmental period in which nutritional and environmental factors play a critical role in the maturation of metabolic, immune, and neuroendocrine systems (1, 2). Traditionally, this period was interpreted only as an interval aimed at ensuring the infant’s physical growth and survival. However, since at least the late twentieth century, growing evidence has advanced and shown that early-life exposures can “program” susceptibility to chronic diseases, including obesity, type 2 diabetes, and cardiovascular disease later in life (1–3).

The contributions of David Barker gave rise to the Developmental Origins of Health and Disease (DOHaD) model, which proposes that adverse gestational conditions such as maternal malnutrition, inflammation, stress, or metabolic dysfunction induce structural and functional adaptations in fetal organs. These adaptations represent survival mechanisms in nature but may generate postnatal metabolic risk (2, 3). Such responses include alterations in angiogenesis, changes in adipocyte differentiation, reduced nephron number, and modifications in neuroendocrine axes, as well as epigenetic changes that may persist into adulthood (1, 2).

In addition to this model, another essential link is the early development of the gut microbiota, a highly dynamic ecosystem that is established from birth and regulates energy metabolism, immune function, systemic inflammation, and glucose–insulin homeostasis (4, 5). The initial colonization process shaped by factors such as mode of delivery, antibiotic exposure, the perinatal environment, and, decisively, breastfeeding defines the microbial trajectory of infancy (4, 6–8). Studies identify early dysbiosis (reduced Bifidobacterium and Lactobacillus and increased pro-inflammatory taxa) as a key factor influencing risks of adiposity, insulin resistance, and lipid metabolism (9–14).

Breastfeeding plays a central role in this process. Beyond its nutritional value, it constitutes a pathway for microbial, immunological, and metabolic transfer (15, 16). Through human milk oligosaccharides (HMOs), immunoglobulins, commensal bacteria, and bioactive metabolites, breastfeeding favorably modulates the infant microbiota, strengthens the intestinal barrier, modulates systemic inflammation, and promotes healthy metabolic regulation (15, 16). Conversely, early cessation of breastfeeding or formula feeding has been associated with specific microbial shifts, particularly a lower abundance of Bifidobacterium a key genus involved in the metabolism of human milk oligosaccharides together with a higher relative abundance of Enterobacteriaceae and members of Clostridia (17, 18). Additionally, these patterns have been linked to an earlier transition toward gut profiles that are less characteristic of breastfeeding and more like those observed at later developmental stages, as well as to variations in microbial metabolite production, including short-chain fatty acids (17–19).

Moreover, epigenetic mechanisms, including DNA methylation, histone modifications, and microRNA regulation, mediate the association between the early nutritional environment and gene expression (1, 2). It has been described that maternal nutrition, dysbiosis, inflammation, and microbial metabolites derived from HMOs and from the fermentation of dietary fiber such as short-chain fatty acids (SCFAs) can modulate these mechanisms, influencing adipogenesis, hypothalamic appetite circuits, insulin sensitivity, and hepatic function (9, 13, 15, 16, 20, 21).

Understanding how the gut microbiota during the first 2 years of life influences early metabolic programming is essential to explain the origins of cardiometabolic diseases later in life. In this regard, the present review integrates the available evidence on the impact of the gut microbiota during the first 2 years of life and its association with the development of type 2 diabetes, arterial hypertension, and obesity, with the purpose of providing an updated, critical, and coherent synthesis of existing findings.

**FIGURE 1:**
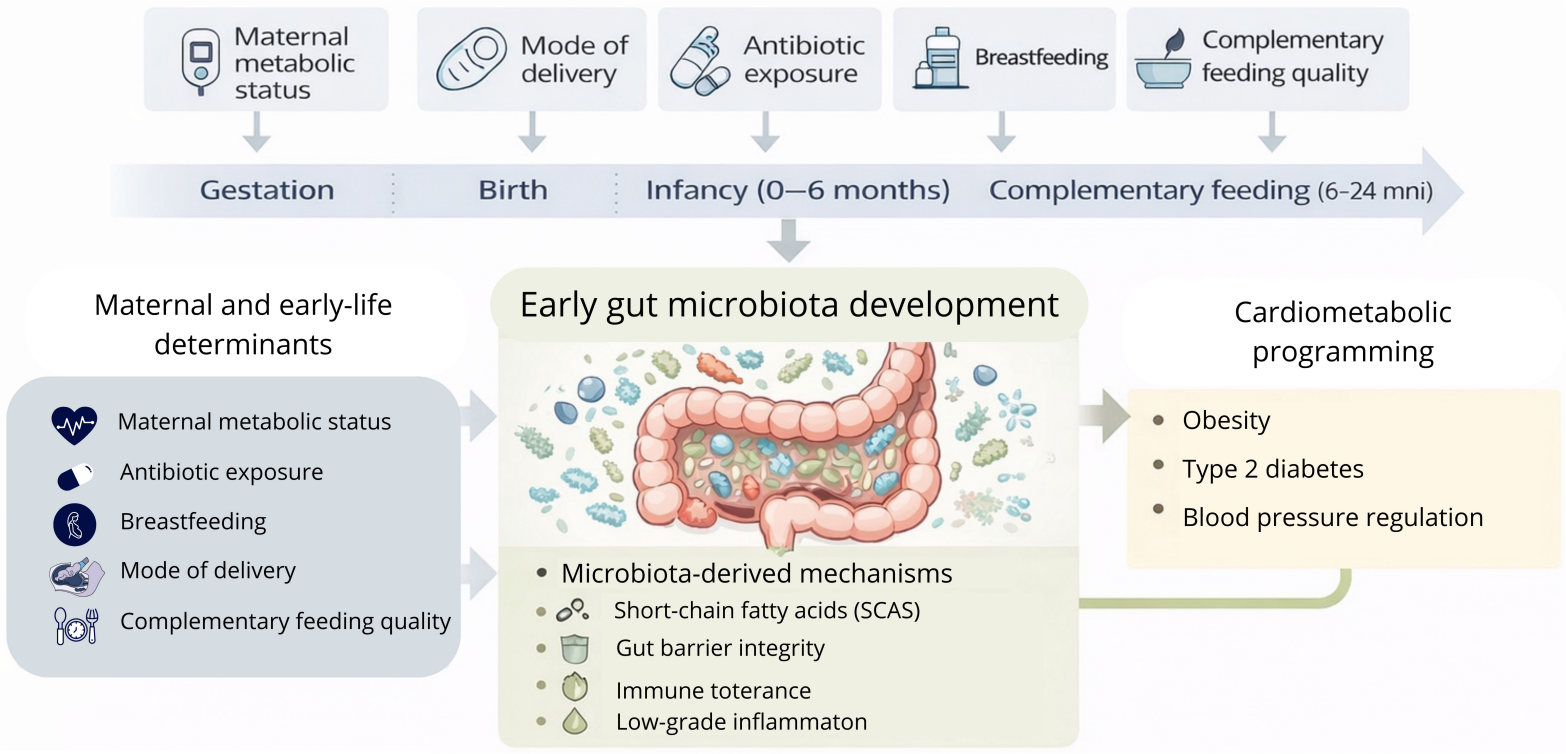
The first 1,000 days as a critical window for gut microbiota-mediated cardiometabolic programming.

This review integrates microbiota-related mechanisms within the DOHaD framework, emphasizing the first 2 years of life as a critical window for cardiometabolic programming (Figure 1).

## 2 The first 1,000 days as a critical window for cardiometabolic programming

This review aimed to describe the influence of the microbiota during the first 2 years of life and its relationship with the development of obesity, type 2 diabetes, and arterial hypertension. In this context, multiple studies recognize that the first 1,000 days from conception to 2 years of age constitute a decisive stage in which immune and metabolic maturation processes consolidate and condition future health (22, 23).

From a chronological perspective, this period integrates a continuous sequence of developmental events that directly influence microbial composition: (i) during the intrauterine and perinatal stage, maternal exposures and metabolic conditions shape the initial ecological context for colonization; (ii) immediately after birth, the newborn gut is typically dominated by early facultative bacteria mainly Enterobacteriaceae and Enterococcus which progressively give way to a more anaerobic and specialized ecosystem; (iii) during breastfeeding, infant-adapted taxa are preferentially established, particularly Bifidobacterium, supporting a more stable community functionally oriented toward the utilization of milk-derived substrates; and (iv) with the introduction of complementary feeding, the microbiota transitions toward higher diversity and a more “adult-like” configuration, characterized by expansion of Firmicutes and Bacteroidetes, alongside increased fermentative and metabolic functions (22, 24).

Overall, this developmental chronology helps explain why the first 1,000 days provide a more informative framework than shorter periods focused exclusively on the neonatal window, and a more appropriate one than broader intervals in which the microbiota progressively stabilizes.

Several studies describe that during this period, factors such as mode of delivery, antibiotic use, and maternal conditions directly influence the initial configuration of the microbiota (22, 25). Added to this are variables such as infant age, early diet, and daycare attendance, which modulate microbial variability during the first months of life (24). Overall, the evidence agrees that microbiota and immunometabolic axis programming begins before birth and continues during the early years, making this early period a fundamental determinant of cardiometabolic risk or protection (22–24).

## 3 Early dysbiosis as an integrating axis of immunometabolic risk

The reviewed studies support that early alteration of the microbiota acts as a central mechanism linking perinatal and environmental exposures with inflammatory responses, metabolic dysregulation, and increased cardiometabolic vulnerability later in life (22–24, 26, 27). At the same time, observational and experimental evidence indicates that conditions such as vaginal delivery, the absence of maternal inflammation, and an adequate nutritional transition favor a state of eubiosis, characterized by greater microbial stability and better immunometabolic maturation (6, 28, 29).

From this perspective, dysbiosis represents an axis through which maternal and early environmental conditions translate into risk trajectories toward obesity, type 2 diabetes, and hypertension (6, 22–24, 26–29).

### 3.1 Influence of perinatal conditions on initial microbial configuration

Regarding perinatal conditions, showed that exposures such as cesarean delivery, maternal inflammation, and early antibiotic use are associated with less favorable microbial profiles from the neonatal period, characterized by a lower abundance of Bifidobacterium and Bacteroides, together with a relative increase in Enterococcus and Proteobacteria, accompanied by functional alterations in metabolic and immune pathways (8). Along the same lines, a recent systematic review on gut microbiota development after cesarean section described patterns recurrently associated with cesarean delivery, including a later enrichment of infant-adapted taxa (particularly Bifidobacterium) and a relative predominance of taxa more frequently linked to early dysbiosis, supporting the reproducibility of these associations across studies (30). In addition, emerging evidence indicates that delivery mode may interact with perinatal determinants such as birth order and sex to modulate neonatal microbial composition, suggesting that cesarean-associated associations are not uniform and may be partially conditioned by concomitant biological and perinatal factors (31). Consistent with this, recent cohort-level findings also report differences in infant gut microbiota composition according to delivery mode during the postnatal period, reinforcing the relevance of cesarean delivery as an early determinant associated with variations in microbial assembly (32). During vaginal delivery, neonates are exposed to maternal microorganisms associated with the vaginal and gut microbiota, including Lactobacillus and infant-adapted anaerobes such as Bifidobacterium and Bacteroides, which support early microbiota maturation and immune training (32, 33).

Concordantly, other studies describe that cesarean delivery, maternal obesity, gestational diabetes, systemic inflammation during pregnancy, perinatal antibiotic exposure, and prematurity are associated with persistent alterations in the neonatal microbiota, reduced early bacterial diversity, and a higher relative abundance of taxa with pro-inflammatory potential (6, 21, 28, 29, 34–37). Beyond these descriptive associations, emerging evidence suggests that early microbial disruptions may be relevant to metabolic programming during critical windows of developmental plasticity. In particular, a reduced abundance of beneficial early-life taxa such as Bifidobacterium and Bacteroides, together with a prolonged dominance of facultative anaerobes, including members of the Enterobacteriaceae family, may interfere with the timely establishment of microbial functions involved in immunometabolic signaling, short-chain fatty acid production, and energy regulation (6, 28, 34, 37). In this context, alterations in microbial maturation trajectories rather than isolated taxonomic shifts may promote low-grade inflammation and impaired immune tolerance, thereby shaping long-term metabolic regulation (6, 29, 34).

From a functional standpoint, these contexts are accompanied by an increase in bacterial genes associated with pro-inflammatory pathways, lipopolysaccharide production, and activation of Toll-like receptors, as well as alterations in metabolic pathways related to fatty acid synthesis and storage (6, 28). In parallel, there is a decrease in microbial functions related to short-chain fatty acid production and maintenance of the intestinal barrier, favoring increased intestinal permeability and systemic bacterial translocation (6, 28, 29) (Table 1).

**TABLE 1:** Main early-life exposure axes and their associated gut microbiota alterations and functional implications.

Exposure axis	Key determinants/context	Associated microbial changes	Functional and developmental implications
Perinatal conditions	Cesarean delivery, prematurity, perinatal antibiotic exposure, maternal inflammation, gestational diabetes	↓ Bifidobacterium, ↓ Bacteroides; ↑ facultative anaerobes (Enterobacteriaceae, Enterococcus, Proteobacteria); reduced early microbial diversity; delayed microbial maturation	Altered immune priming; increased pro-inflammatory tone; impaired establishment of immune tolerance; disruption of early immunometabolic signaling
Exclusive breastfeeding	Human milk exposure during early infancy; availability of human milk oligosaccharides (HMOs)	Enrichment of infant-adapted taxa (Bifidobacterium spp., Lactobacillus); stable early community structure; dominance of HMO-utilizing bacteria	Enhanced short-chain fatty acid production; improved gut barrier integrity; immune education; support of balanced immunometabolic maturation
Formula feeding/early weaning	Partial or absent breastfeeding; early formula introduction	Reduced Bifidobacterium predominance; earlier transition toward adult-like microbiota; increased community instability	Altered microbial succession; reduced microbial specialization; potential disruption of early immune–metabolic interactions
Complementary feeding	Timing and quality of solid food introduction; fiber-rich vs. Western-type diets	Increased microbial diversity; expansion of Firmicutes and Bacteroidetes; enrichment of carbohydrate-fermenting taxa; diet-dependent shifts in SCFA-related profiles	Modulation of microbial succession; influence on metabolic outputs; effects dependent on dietary quality and concurrent exposures
Childhood obesity–related microbial patterns	Accelerated early growth trajectories; cumulative early-life exposures	Reduced infant-adapted bacteria (notably Bifidobacterium); increased Proteobacteria/Enterobacteriaceae; functional signals related to LPS and altered SCFA profiles	Low-grade inflammation; metabolic endotoxemia signals; altered energy regulation; obesity risk as an ecological phenotype rather than a single taxonomic signature

### 3.2 Early microbiota and risk of type 2 diabetes

With respect to type 2 diabetes, most studies in infants and children under 2 years show that microbial profiles with lower diversity, reduced Bifidobacterium and Lactobacillus, and increased Firmicutes and Proteobacteria are associated with early markers of glycemic alteration (12, 23, 26, 27, 36, 38–40).

In the Latin American context, studies conducted in Brazil report that schoolchildren with lower levels of Bifidobacterium more frequently present elevated glucose values, reinforcing its role in pediatric metabolic regulation (26). Complementarily, research in pregnant women with obesity or gestational diabetes shows maternal microbiotas depleted in beneficial genera, which could facilitate transmission of a less balanced microbial ecosystem to the newborn (21, 34–36, 38, 41–49).

Interventions aimed at microbial modulation during early life have primarily focused on probiotic supplementation (single strains or multi-strain formulations) administered to breastfed infants or very young infants. For instance, early supplementation with Bifidobacterium infantis in breastfed infants has been associated with persistent colonization over time and with an infant gut microbiota profile enriched in infant-adapted bifidobacteria (50). In parallel, clinical studies evaluating infant formulas containing strains such as *Bifidobacterium animalism* ssp. lactis or *Lactobacillus salivarius* have reported detectable shifts in gut bacterial composition during the intervention period, together with acceptable safety profiles and no major adverse effects on growth outcomes (51). Other pilot trials in infants using human milk–derived strains (e.g., Bifidobacterium breve) also support feasibility and short-term modulation of gut microbial profiles, although findings vary depending on the strain, dose, feeding mode, and baseline microbiota (52). Beyond probiotics, observational studies in mother–infant dyads highlight that metabolic conditions such as gestational diabetes may influence the maternal–infant microbial axis through altered human milk oligosaccharide patterns, which may hinder colonization by beneficial taxa and affect immune tolerance–related pathways in the neonate (53, 54). Overall, while these approaches suggest that targeted microbial modulation is biologically plausible, the field remains limited by heterogeneity in study designs, outcomes, sequencing/analytical methods, and follow-up duration, which restricts firm conclusions regarding clinical applicability and underscores the need for standardized, strain-specific, outcome-oriented trials before translation into routine practice (27, 50–58).

### 3.3 Gut microbiota and early regulation of blood pressure

In contrast to obesity and type 2 diabetes, the evidence linking early-life microbiota development to blood pressure regulation remains comparatively limited, particularly during the first 2 years of life when microbial succession is highly dynamic and strongly shaped by early-life exposures (2, 4, 5). This disparity is important within the developmental origins of health and disease framework, as the first 1,000 days represent a critical window during which early biological adaptations may influence long-term cardiometabolic trajectories (1–3). However, while microbiome-mediated “early programming” of blood pressure is biologically plausible, the pediatric literature is currently less mature than that supporting adiposity-related outcomes, and conclusions should be interpreted with caution (2, 4, 14).

At present, the most consistent evidence supports associations between gut microbiota-related metabolic signals and blood pressure abnormalities in pediatric populations with underlying renal or cardiometabolic vulnerability, rather than in healthy infant cohorts. In these settings, microbial metabolites particularly short-chain fatty acids (SCFAs) have been proposed as candidate mediators linking microbial ecology with vascular and inflammatory regulation (59). For example, propionate- and butyrate-related profiles have been discussed in relation to blood pressure abnormalities in children with congenital anomalies of the kidney and urinary tract (59). Nevertheless, such findings cannot be directly extrapolated to infants within the first 1,000 days, as disease-related physiology and clinical interventions may independently influence microbial composition and metabolite profiles.

From a taxonomic perspective, SCFA-producing taxa such as Faecalibacterium, Roseburia, Eubacterium, and Bacteroides are often framed as potentially beneficial contributors to immune and barrier homeostasis during infancy and early childhood (4, 11, 14). Yet, most available studies remain correlational, and robust longitudinal pediatric cohorts linking early microbial features to subsequent blood pressure trajectories as a predefined clinical endpoint are scarce (4, 5, 14). Moreover, early-life dysbiosis is shaped by multiple programming factors including delivery mode, feeding practices, and inflammatory exposures which may introduce residual confounding and complicate causal inference when blood pressure is assessed as an outcome (4–6, 8). Overall, current data more strongly support microbiome-related hypotheses for early blood pressure regulation as plausible mechanistic frameworks than as established predictive signatures during the first 1,000 days (4, 14).

Therefore, future longitudinal studies beginning in early infancy are needed to clarify whether early microbial configurations and metabolite-related profiles predict later blood pressure trajectories, rather than reflecting secondary microbial shifts associated with underlying disease states or clinical exposures (4, 5, 14, 59).

#### 3.3.1 Exclusive breastfeeding and protective infant gut microbiota trajectories

Exclusive breastfeeding is consistently described as a key determinant of early-life gut microbiota assembly, promoting microbial ecosystems that are functionally specialized and potentially protective against later cardiometabolic risk (16, 19, 60, 61). Importantly, evidence from pediatric cohorts comparing feeding modes shows that breastfed infants develop gut microbial profiles enriched in infant-adapted taxa, whereas formula-fed infants exhibit earlier transitions toward more “adult-like” configurations and distinct microbial maturation patterns, which have been linked to differences in growth trajectories during infancy (16, 19). A central hallmark of breastfeeding-associated microbial development is the preferential enrichment of Bifidobacterium, supported by the availability of human milk oligosaccharides (HMOs) as selective substrates, which strengthens priority effects and supports stable early colonization (60–62). In contrast, studies comparing exclusively breastfed versus formula-fed infants describe reduced Bifidobacterium predominance and altered community structure in formula-fed groups, alongside differences in growth-related phenotypes (19). Cohort-level longitudinal evidence further suggests that early microbiota maturation patterns during the first year closely aligned with early feeding exposures are associated with subsequent BMI trajectories and rapid growth phenotypes that increase the probability of later overweight risk (9, 17).

Beyond taxonomy, breastfeeding-driven microbial ecosystems may be clinically meaningful because they support functions linked to SCFA production, immune tolerance, and barrier integrity mechanisms repeatedly implicated in pediatric metabolic homeostasis. Conversely, deviations from infant-adapted succession patterns during this window more frequently observed with early formula feeding have been associated with obesity-related microbial signatures reported in pediatric cohorts and systematic syntheses (9, 23, 27, 63). Overall, these data support that breastfeeding is not only a nutritional exposure but also a microbiome-shaping factor with potential downstream relevance for long-term metabolic health, reinforcing its role as a preventive strategy within the first 1,000 days (9, 16, 17, 19, 23, 27, 60, 61, 64).

Exclusive breastfeeding supports an infant-adapted gut microbiota characterized by a strong enrichment of Bifidobacterium and HMO-driven metabolic functions, which are consistently linked to improved microbial stability and immune education during early life (16, 19, 60–62). Clinically, this may translate into a lower pro-inflammatory tone and a more favorable metabolic programming trajectory, whereas formula feeding has been associated with earlier microbiota maturation, reduced Bifidobacterium predominance, and distinct growth-related phenotypes, including differences in weight trajectories (19, 65). Although long-term cardiometabolic outcomes still require stronger prospective validation, current evidence supports breastfeeding-associated microbial patterns as a biologically plausible protective pathway against low-grade inflammation and later metabolic vulnerability, reinforcing breastfeeding as a microbiome-relevant preventive strategy within the first 1,000 days (9, 16, 17, 19, 63, 66–68).

## 4 Complementary feeding

Complementary feeding represents a major ecological “inflection point” in early-life gut microbiota development, because it introduces new substrates that can rapidly reshape community structure and metabolic outputs. In longitudinal infant studies, the timing of complementary feeding has been associated with measurable differences in microbiota diversity/composition and SCFA concentrations across the first year, supporting the concept that dietary transitions can modify succession during a highly plastic window (69). From this perspective, the shift from an HMO-driven ecosystem toward a diet-driven ecosystem is expected to promote higher diversity and increased representation of taxa involved in complex carbohydrate fermentation, commonly reflected as expansion of Firmicutes and Bacteroidetes alongside enrichment of fermentative functions (9, 17, 23, 27). However, the direction and clinical meaning of these shifts are not uniform across studies, largely because complementary feeding varies widely in quality (fiber-rich vs. Western-type), macronutrient profile, and co-exposures (formula use, antibiotics, perinatal factors), complicating direct comparisons (23, 27, 70, 71).

Across cohorts and syntheses, higher fiber/plant-based patterns during complementary feeding are generally interpreted as favoring SCFA-producing networks, whereas more “Western” complementary feeding patterns may promote pro-inflammatory configurations and metabolic dysregulation signals (23, 27, 63). This interpretation is supported by pediatric obesity data showing that SCFA-related signatures and microbial configurations differ in children with obesity, and SCFA–microbiota relationships have been described in obese pediatric populations (64). At the same time, caution is warranted: SCFAs can reflect both beneficial fermentation and increased energy harvest, and many observational datasets cannot disentangle whether microbial changes precede adiposity or are partly shaped by early growth trajectories and dietary choices (“reverse causality”) (9, 17, 23, 64). Moreover, infant formula composition itself can accelerate microbiota maturation toward more “adult-like” patterns and is associated with differences in weight gain velocity and weight status, which can confound complementary-feeding effects when formula exposure overlaps with the complementary feeding window (65).

From a clinical–ecological standpoint, the most consistent microbiome-relevant signal is that complementary feeding can either support or disrupt healthy succession depending on dietary quality and concurrent exposures. Studies integrating diet with microbiota/metabolome profiles at 1 year indicate that diet–microbiota relationships are already detectable at this stage and may align with metabolic readouts, reinforcing clinical relevance but also highlighting heterogeneity across populations and dietary contexts (71). Therefore, while the literature supports complementary feeding as a modifiable lever for shaping microbial succession, stronger causal inference will require harmonized dietary exposure definitions, better control for breastfeeding/formula dynamics, and more studies linking complementary-feeding–related microbial shifts to harder cardiometabolic outcomes beyond BMI trajectories (9, 17, 23, 27, 63–71).

### 4.1 Childhood obesity and gut microbiome signatures

Pediatric obesity represents a growing global health challenge, closely associated with increased cardiometabolic risk and long-term adverse outcomes. (72) Emerging evidence supports that the gut microbiota plays a relevant role in obesity pathogenesis by influencing host metabolism, energy homeostasis, and systemic inflammation; however, the available pediatric clinical evidence remains heterogeneous and robust data are still limited, warranting cautious interpretation.

During the first 1,000 days of life, the gut undergoes a phase of high ecological plasticity in which the microbiota is shaped through a process of microbial succession that is highly sensitive to maternal and postnatal exposures (2). During this period, subtle deviations in early colonization may be amplified and consolidated into distinct metabolic trajectories, contributing to the “programming” of future cardiometabolic risk (2). In the context of childhood obesity, the available evidence suggests that risk does not depend on a single causal microorganism, but rather on the progressive acquisition of a microbial and functional configuration that favors low-grade inflammation, greater efficiency of energy extraction, and early signals of immunometabolic dysfunction (23, 63). Therefore, the concept of an “obesogenic microbiota” should be understood as a complex ecological phenotype rather than a universal taxonomic signature (23, 63).

Longitudinal studies have shown that children with accelerated growth trajectories or greater early weight gain exhibit distinct microbial compositions from the first year of life, supporting the hypothesis that part of the risk is established before obesity becomes clinically evident (2). However, although repeated associations have been reported between obesity and shifts in major phyla such as Firmicutes and Bacteroidetes, these findings are not consistently replicated across cohorts, likely due to differences in sampling age, prevailing diets, environmental exposures, geographic context, and methodological variability, which calls for a critical interpretation of so-called “obesity microbial signatures” (9, 23, 63). It is important to highlight that phylum-level patterns (including signals related to Firmicutes/Bacteroidetes) show marked inconsistency across different pediatric settings, which limits their reliability as stand-alone biomarkers and supports the need for higher-resolution and function-oriented interpretations (9, 23, 63). Accordingly, a more robust approach is to interpret the evidence by emphasizing recurrent ecological and functional patterns rather than relying on phylum-level proportions as universal markers (9, 23, 63).

Within this framework, a higher obesity risk tends to be associated with a shift from an ecosystem dominated by infant-adapted bacteria toward less specialized communities, with lower stability and greater pro-inflammatory potential (23, 63). A particularly relevant finding is the reduction or deficit of Bifidobacterium in infants, given its central role in the utilization of human milk oligosaccharides, immunomodulation, and the formation of cross-feeding networks that support beneficial communities during infancy (22, 60, 62). Recent evidence also indicates that Bifidobacterium community assembly depends not only on breastfeeding, but also on the availability of specific oligosaccharides and priority effects in microbial assembly, which may condition subsequent intestinal maturation (60, 62). In parallel, early dysbiosis signals have been described in formula-fed infants compared with those exclusively breastfed, with differences associated with growth status (19, 65). Consistent with this, infants with dysbiosis may exhibit an expansion of opportunistic taxa, particularly Proteobacteria/Enterobacteriaceae, which is interpreted as an ecosystem with higher inflammatory potential and disruption of intestinal homeostasis (19, 22, 63).

The available evidence indicates that the relative increase in gram-negative taxa is relevant due to their association with lipopolysaccharides (LPS), as exposure to gut-derived LPS has been linked to metabolic endotoxemia and systemic low-grade inflammation (73). Nevertheless, the role of short-chain fatty acids (SCFAs) requires cautious interpretation. SCFAs have traditionally been viewed as beneficial mediators because of their contribution to gut barrier health and immune regulation; however, in obesity, certain fermentative profiles may also reflect greater efficiency of energy extraction (9, 63, 64). This helps explain why some studies report associations between childhood obesity, shifts in SCFA-producing taxa, and differences in SCFA concentrations, whereas others describe divergent results depending on dietary context and child development (9, 63, 64). Therefore, SCFAs should not be presented as a linear biomarker that is uniformly protective or harmful, but rather as a metabolic signal whose impact depends on age, feeding patterns, overall community composition, and growth dynamics (9, 63, 64). In addition, many pediatric datasets cannot fully disentangle whether microbial features precede adiposity development or partially reflect early growth patterns and dietary exposures (“reverse causality”), which further complicates causal interpretation (9, 23, 63).

The development of an obesogenic microbiota during the first 1,000 days is typically the result of cumulative exposures rather than a single event. Among the most relevant postnatal factors are feeding mode, timing of complementary feeding introduction, and antibiotic exposure (19, 65, 69). The macronutrient composition of infant formula has been shown to produce differences in microbial maturation, and these variations have been associated with weight gain velocity and weight status (65). Likewise, the timing of complementary feeding has been associated with microbiota diversity and composition during the first year, as well as with SCFA concentrations, suggesting a pathway through which dietary transitions may modulate future metabolic trajectories (69). In addition, early antibiotic exposure has been associated with a higher risk of childhood overweight or obesity, and part of this association appears to be mediated by alterations in the gut microbiota (10, 74, 75). Cohort studies have reported that antibiotic exposure in early life increases overweight/obesity risk in relation to dysbiosis, and measurable microbiota changes have been described following systemic antibiotic use in infants, reinforcing the plausibility of this mechanism (76, 77). In this scenario, antibiotic exposure represents a potent disruptor during a period in which the community has not yet developed ecological resilience, facilitating shifts toward opportunistic taxa and reducing microorganisms that are key for immune maturation (10, 76, 77).

Maternal factors may also play a structuring role in initial colonization and metabolic programming. Evidence related to gestational diabetes has shown alterations in both maternal microbiota and early infant microbial seeding, with potential implications for later body mass index (BMI) (13, 36, 49). Consistent with this, gestational diabetes has been associated with a distinct pattern of early microbiota acquisition, and microbial changes may act as mediators in the increased infant BMI observed in this context (36, 49). Additionally, alterations in human milk oligosaccharides and lipid components in mothers with gestational diabetes have been reported to hinder colonization by beneficial bacteria and influence the development of neonatal immune tolerance, reinforcing the relevance of the maternal–milk–infant microbiota axis (47, 54). In a complementary way, maternal dietary interventions with higher carbohydrate complexity during gestation in gestational diabetes have been associated with increased bifidobacteria and changes in early infant microbiota acquisition, suggesting a potentially modifiable preventive pathway (78).

In summary, the first 1,000 days represent a critical period because they encompass initial gut ecosystem establishment, the selective influence of breastfeeding, and the dietary transition toward more complex foods, determining whether the microbiota evolves toward a more stable trajectory or toward configurations with greater pro-inflammatory and obesogenic potential (2). Although specific taxonomic signatures vary across studies, there is biological coherence in the idea that the loss of beneficial infant bacteria particularly Bifidobacterium together with increased dysbiosis and endotoxemia signals such as Proteobacteria/LPS, may contribute to the early programming of obesity risk (19, 22, 63, 73). However, methodological heterogeneity, dietary differences and geographic context, and the predominance of observational designs require cautious interpretation of these findings, highlighting that the greatest contribution of the literature lies in the convergence of mechanisms (barrier function, inflammation, and immunometabolic regulation) rather than in a single universal microbial signature (9, 23, 63).

## 5 Integrative synthesis and preventive projection

In summary, the analyzed evidence supports that early-life microbiota disruption represents a transversal mechanism connecting the first 1,000 days with later cardiometabolic risk, including obesity, type 2 diabetes, and arterial hypertension (22, 24, 26, 27, 63, 64, 79, 80). However, it is important to acknowledge that the strength of evidence is not uniform across outcomes: associations are most consistent for obesity and metabolic dysregulation, while links to blood pressure regulation remain less robust and more context-dependent, with a predominance of indirect or disease-specific pediatric data.

Importantly, prevention strategies during this critical window should move beyond general statements and include concrete, microbiome-relevant actions: (i) optimization of maternal metabolic health before and during pregnancy (e.g., weight management, gestational diabetes prevention/control, and reduction of inflammatory exposures) to favor more stable maternal–infant microbial transfer (21, 35, 36, 79); (ii) promotion of vaginal delivery when clinically feasible, given its role in early microbial seeding and enrichment of infant-adapted anaerobes (6, 24); (iii) strengthening breastfeeding practices to sustain Bifidobacterium-dominant ecosystems and support gut maturation through human milk–microbiome interactions (9, 22, 26, 35); (iv) ensuring appropriate complementary feeding patterns (timing and dietary quality) that support microbial succession and reduce obesogenic signatures (9, 17, 23, 27, 64, 70); and (v) prudent antibiotic use in mothers and infants, as early antimicrobial exposure is consistently linked to altered microbial trajectories and reduced resilience (7, 77). Notably, heterogeneity across cohorts driven by geographic context, sequencing pipelines, dietary exposures, and variable outcome definitions partly explains inconsistencies in reported microbial “signatures,” reinforcing the need for standardized longitudinal designs and harmonized analytical frameworks. Finally, emerging but still debated approaches aimed at restoring microbial exposure after cesarean delivery such as probiotic supplementation or microbiota-directed interventions remain under evaluation and require standardized protocols and long-term safety evidence before routine implementation (37, 80–84).

## 6 Implications

### 6.1 Clinical implications

The findings of this review suggest that the first 2 years of life constitute a particularly sensitive window for cardiometabolic programming, in which the gut microbiota functions as an integrating axis of maternal, perinatal, and early environmental exposures. In clinical practice, this reinforces the need to intervene early, prioritizing gestation and the first postnatal months, when intestinal colonization is more plastic and, therefore, more susceptible to modulation.

Specifically, prenatal and neonatal care should incorporate a preventive approach focused on: (i) optimizing maternal metabolic and nutritional status (obesity, gestational diabetes, and glycemic control), (ii) strategies for prudent antibiotic use in the mother and newborn (antibiotic stewardship), and (iii) strengthening effective breastfeeding support, given its role in establishing microbial profiles considered more favorable. At the same time, the results support the relevance of early monitoring of metabolic risk indicators (adiposity and glycemic control), whereas, for arterial hypertension, evidence in pediatric populations remains insufficient for conclusive recommendations; therefore, interpretive caution and longitudinal follow-up are warranted.

### 6.2 Implications for health policy

From a public health perspective, the results indicate that policies focused on the first 1,000 days should be structured around three articulated axes: pregnancy and prenatal care, breastfeeding, and complementary feeding. Nevertheless, the evidence suggests that the greatest “programming impact” is concentrated in gestation and the early neonatal period; therefore, population-level strategies should intensify actions in this critical segment to reduce persistent risk trajectories, consistent with the DOHaD framework.

This implies strengthening policies that: (1) ensure timely access to prenatal care including components of maternal metabolic health (screening and management), (2) promote institutional and community conditions to sustain breastfeeding (professional support, enabling environments, and social protection), (3) regulate and provide training on the rational use of antibiotics in the perinatal period, and (4) strengthen high-quality complementary feeding programs as preventive continuity. Finally, the review supports the need to invest in longitudinal and regional research, especially to clarify mechanisms and early associations with hypertension, and thus translate microbial evidence into more specific recommendations applicable to local contexts.

## 7 Future windows and perspectives for intervention

The evidence synthesized in this review suggests that the most promising interventions for cardiometabolic prevention should be strategically situated within the first 1,000 days, with particular emphasis on gestation and the first postnatal months, when the gut microbiota shows greater plasticity. In this framework, a priority future window is the design and implementation of integrated mother–child interventions that combine maternal nutritional optimization, metabolic control during pregnancy, and early breastfeeding support strategies.

Likewise, a relevant field is emerging for targeted microbial modulation interventions, including the rational use of probiotics, prebiotics, and synbiotics, as well as promotion of complementary feeding patterns rich in fiber and minimally processed foods, adapted to sociocultural contexts. However, these strategies require validation through longitudinal studies and well-designed clinical trials that evaluate not only changes in microbiota composition, but also medium- and long-term metabolic outcomes.

Finally, future intervention lines should integrate preventive medicine and public health approaches, incorporating early microbial markers as potential risk stratification tools. The development of policies based on this evidence could enable a shift from reactive models toward early preventive strategies, with sustained impact on reducing obesity, type 2 diabetes, and arterial hypertension across the life course.
